# Pulse Pressure, Cognition, and White Matter Lesions: A Mediation Analysis

**DOI:** 10.3389/fcvm.2021.654522

**Published:** 2021-05-04

**Authors:** Jiabin Zang, Jian Shi, Jianwen Liang, Xiaocong Zhang, Wenbin Wei, Chun Yao, Xiaodong Zhuang, Guifu Wu

**Affiliations:** ^1^Department of Cardiology, The Third Affiliated Hospital of Sun Yat-sen University, Guangzhou, China; ^2^Department of Cardiology, The Eighth Affiliated Hospital of Sun Yat-sen University, Shenzhen, China; ^3^Guangdong Innovative Engineering and Technology Research Center for Assisted Circulation, Shenzhen, China; ^4^Department of Cardiology, The First Affiliated Hospital of Sun Yat-sen University, Guangzhou, China; ^5^NHC Key Laboratory of Assisted Circulation, Sun Yat-sen University, Guangzhou, China

**Keywords:** pulse pressure, cognition, white matter lesions, sprint, mediation analysis

## Abstract

This study aimed to investigate the effects of pulse pressure (PP) on cognition and the role of white matter lesions (WMLs) in mediating this association. We enrolled 3,009 participants from the SPRINT-MIND study. Of those, 755 participants underwent brain magnetic resonance imaging. Cognitive tests were summarized in five cognition domains, including global cognition, executive function, attention, memory, and language. Multiple linear regression models were employed to analyze PP in association with cognition, and mediation analysis was applied to determine the role of WMLs in the association between PP and cognition. We found that PP was negatively linearly associated with global cognition (β = −0.048, *P* = 0.008), executive function (β = −0.014, *P* = 0.040), attention (β = −0.013, *P* = 0.035), memory (β = −0.021, *P* = 0.045), and language (β = −0.020, *P* = 0.001), respectively. Furthermore, PP was not significantly associated with brain component volume changes, except for WMLs (β = 0.029, *P* = 0.044). Additionally, mediation analysis showed that increased WML volume contributed to 10.8% of global cognition, 9.5% of executive function, 10.6% of memory, and 7.2% of language decline associated with PP. Exposure to higher PP levels was associated with poor cognitive performance, and WMLs partially moderated the influence of PP on cognition.

## Introduction

Most previous studies have shown that elevated blood pressure (BP) exacerbates cognitive impairment (1–3). Cognitive decline occurs mostly in middle-aged and older populations, and one of the characteristics of BP in this age group is its tendency toward high systolic blood pressure (SBP) and low diastolic blood pressure (DBP). Therefore, the role of elevated pulse pressure (PP) in the cognitive decline process needs to be investigated.

The association between PP and cognition remains controversial. To our knowledge, a community-based longitudinal study is the first to demonstrate that higher PP is associated with increased risk for Alzheimer's disease and dementia (4). A secondary analysis of the hypertension in the very elderly trial (HYVET) indicated that wider PP may increase the risk of dementia (5). Similar results were reported in other studies (6–8). In contrast, a few studies suggested that higher PP is not independently associated with cognitive decline (9, 10).

In addition, increased brachial PP is an age-independent factor associated with white matter lesions (WMLs) in elderly individuals, while the association between WMLs and cognition is already established (11, 12). A few studies are currently available on the effect of WMLs on the association between PP and cognition domains in stroke-free adults with hypertension. Therefore, in the present study, we assessed whether PP was associated with cognition using Systolic BP Intervention Trial-Memory and cognition IN Decreased hypertension (SPRINT-MIND) baseline data and explored the potential mechanism by which WMLs moderate the association between PP and cognition.

## Methods

### Study Population

This was a cross-sectional study of SPRINT-MIND data obtained from the National Heart, Lung, and Blood Institute. SPRINT was a multicenter, randomized controlled trial that examined whether intensive BP treatment (SBP <120 mm Hg) would reduce the risk of cardiovascular events and total mortality compared with standard BP treatment (SBP <140 mm Hg) among 9,361 participants aged ≥50 years with hypertension (SBP of 130 to 180 mm Hg). The detailed acceptance criteria and methods have been described in the previous SPRINT design study (13). A subset of 3,009 participants who answered cognitive function questionnaires and 755 participants who underwent brain MRI scan at baseline were enrolled in the MIND cohort (Supplementary Figure 1).

### Blood Pressure Measurement

BP was measured at each clinic visit after a rest period using an automated device that reduced potential for observer biases. PP was calculated by SBP minus DBP at baseline. PP was analyzed as a continuous variable and categorical variable, respectively.

### Cognitive Tests

The MIND screening battery included Montreal Cognitive Assessment, Logical Memory Test, and Digits Symbol Coding Test, while the MIND extended battery included Hopkins Verbal Learning Test, Trail Making Test, Digit Span Test, Boston Naming Test, and Category Fluency Test—Animals. These cognitive tests were summarized to five specific major cognition domains, including global cognition, executive function, memory, attention, and language (Supplementary Table 1). Individual test results were standardized as z scores added to develop summary cognition domain scores. Lower scores indicate poor cognitive performance.

### Brain Magnetic Resonance Imaging

In the SPRINT-MIND study, 755 participants completed brain MRI at baseline. Several 3.0-T MRI scanner models from manufacturers (Siemens, Philips, and GE Healthcare) were used to perform the brain MRI. At least one trained and certified technician was responsible for MRI quality control at each participating field center. The image data were transmitted from the field center to the MRI reading center at the University of Pennsylvania for review. Using a label fusion method, the brain tissue was divided into several anatomical regions of interest (14). The WMLs were characterized from fluid-attenuated inversion recovery and T1-weighted images by applying a deep learning-based segmentation technique (15).

### Statistical Analysis

All variables at SPRINT-MIND baseline were summarized using standard descriptive statistics, and stratified by PP quartile.

Using a multivariate linear regression model, we examined the association of PP with cognitive tests and brain MRI variables. Individual tests results were standardized as z scores. In addition, we adjusted for covariates, including age, gender, race, education, smoking, drinking, body mass index, cardiovascular disease (CVD), cholesterol, fasting plasma glucose, estimated glomerular filtration rate, and medication use (statin, aspirin, and antihypertensive). Analyses involving brain MRI variables were additionally adjusted for scanner type, intracranal volume, and brain volume.

Mediation analysis was conducted to characterize the cognitive effects of PP that could be explained by WMLs. That is, analyses were used to identify and explain the mechanism pathways that underlie an observed relationship between an independent variable (PP) and outcome variables (cognition parameters) via a mediator (brain MRI variables). It allows estimation of the direct and indirect effects and the proportion mediated. The proportion can be calculated by dividing the indirect effect by the total effect. In this study, all analyses were performed using SPSS software, version 22 (Chicago, IL, USA).

## Results

### Participant Characteristics

At the SPRINT-MIND study baseline, 3,009 participants (of whom 755 underwent brain MRI) had available PP values and completed the MIND questionnaires including dementia screening and extended cognitive battery. The mean age was 68.6 ± 8.7 years, 1,103 (36.7%) were female, 1,798 (59.8%) were white, and 2,226 (74.8%) had advanced education. The mean PP level was 61.64 ± 14.62 mm Hg. There were 610 (20.3%) participants with CVD history and 939 (31.2%) participants with CKD history.

Compared with participants with low or normal PP (PP ≤ 60 mm Hg), participants with PP > 60 mm Hg were more likely to be older, female, former smoker, and had a higher CVD risk score and CKD history (Table 1, Supplementary Table 2).

**Table 1:** Baseline characteristics of Systolic BP Intervention Trial-Memory and cognition IN Decreased hypertension (SPRINT-MIND) participants classified by pulse rressure (PP) quartile.

**Characteristic**	**Total (*n* = 3,009)**	**PP, mm Hg**				
		**Quartile 1 (*n* = 793)**	**Quartile 2 (*n* = 739)**	**Quartile 3 (*n* = 716)**	**Quartile 4 (*n* = 761)**	***P*-value**
Age, year	68.6 ± 8.7	64.0 ± 7.1	67.0 ± 7.9	70.0 ± 8.1	73.9 ± 8.4	<0.001
Age ≥75 years, *n* (%)	843 (28.0)	69 (8.7)	146 (19.8)	226 (31.6)	402 (52.8)	<0.001
Female, *n* (%)	1,103 (36.7)	270 (34.0)	230 (31.1)	271 (37.8)	332 (43.6)	<0.001
Race, *n* (%)						<0.001
White	1,798 (59.8)	428 (54.0)	418 (56.6)	431 (60.2)	521 (68.5)	
Black	894 (29.7)	301 (38.0)	229 (31.0)	200 (27.9)	164 (21.6)	
Hispanic	254 (8.4)	56 (7.1)	71 (9.6)	67 (9.4)	60 (7.9)	
Other	63 (2.1)	8 (1.0)	21 (2.8)	18 (2.5)	16 (2.1)	
Education level^a^, *n* (%)						0.187
Low	46 (1.5)	14 (1.8)	8 (1.1)	7 (1.0)	17 (2.3)	
Intermediate	704 (23.7)	195 (24.9)	184 (25.1)	164 (23.2)	161 (21.4)	
High	2,226 (74.8)	574 (73.3)	540 (73.8)	536 (75.8)	576 (76.4)	
Smoking status, *n* (%)						0.001
Never	1,324 (44.0)	351 (44.3)	326 (44.1)	318 (44.4)	329 (43.2)	
Former smoker	1,319 (43.8)	315 (39.7)	314 (42.5)	331 (46.2)	359 (47.2)	
Current smoker	362 (12.0)	127 (16.0)	98 (13.3)	66 (9.2)	71 (9.3)	
FRS, %	19.9 ± 10.8	14.7 ± 8.0	18.6 ± 9.8	20.4 ± 9.7	26.0 ± 12.1	<0.001
CVD history, *n* (%)	610 (20.3)	131 (16.5)	126 (17.1)	146 (20.4)	207 (27.2)	<0.001
CKD history, *n* (%)	939 (31.2)	209 (26.4)	203 (27.5)	215 (30.0)	312 (41.0)	<0.001
BMI, kg/m^2^	29.8 ± 5.6	31.1 ± 6.0	30.0 ± 5.6	29.6 ± 5.6	28.5 ± 5.1	<0.001
SBP, mm Hg	138.9 ± 16.0	126.0 ± 11.1	134.8 ± 11.1	141.7 ± 11.5	153.6 ± 15.1	<0.001
DBP, mm Hg	77.2 ± 11.8	81.0 ± 10.0	78.8 ± 10.9	76.5 ± 11.5	72.5 ± 12.8	<0.001
LDL-c, mg/dl	111.7 ± 35.4	114.7 ± 35.3	112.0 ± 33.8	110.6 ± 36.0	109.5 ± 36.4	0.026
HDL-c, mg/dl	53.4 ± 14.5	51.3 ± 13.5	52.5 ± 14.2	54.6 ± 14.6	55.5 ± 15.5	<0.001
Glucose, mg/dl	99.2 ± 13.7	98.8 ± 15.0	99.3 ± 11.9	99.0 ± 13.0	99.9 ± 14.5	0.461
eGFR, ml/min/1.73 m^2^	70.4 ± 20.8	72.2 ± 20.8	72.0 ± 19.8	71.2 ± 20.8	66.2 ± 21.4	<0.001
Medication use, *n* (%)						
Statin	1,313 (44.0)	322 (40.8)	328 (44.7)	329 (46.3)	334 (44.4)	0.177
Aspirin	1,566 (52.2)	382 (48.2)	370 (50.2)	391 (54.8)	423 (55.9)	0.006
Antihypertensive agents	2,741 (91.1)	718 (90.5)	648(90.7)	705 (92.6)	2,122 (91.9)	0.388

*Note. Values are mean ± SD or number (%).*

*Quartile 1, PP ≤ 51 mm Hg; Quartile 2, 51 mm Hg < PP ≤ 60 mm Hg; Quartile 3, 60 mm Hg < PP ≤ 70 mm Hg; Quartile 4, PP > 70 mm Hg.*

a *Education level including low, below high school graduate; intermediate, high school graduate; high, beyond high school.*

*SPRINT, Systolic Blood Pressure Intervention Trial; MIND, Memory and cognition In Decreased hypertension; FRS, Framingham Risk Score; CVD, cardiovascular disease; CKD, chronic kidney disease; SBP, systolic blood pressure; DBP, diastolic blood pressure; PP, pulse pressure; BMI, body mass index; LDL-c, low-density lipoprotein cholesterol; HDL-c, high-density lipoprotein cholesterol; eGFR, estimated glomerular filtration rate*.

### Association Between Pulse Pressure and Cognition

Unadjusted performance comparisons on individual cognitive tests by PP categories are shown in Table 2. For the mean scores of all cognitive tests, statistical differences were observed across the PP strata.

**Table 2:** Performance on cognitive tests by PP quartile in SPRINT-MIND participants.

**Cognitive Tests**	**PP, mm Hg (*****n*** **=** **3,009)**				***P*-value**
	**Quartile 1 (*n* = 793)**	**Quartile 2 (*n* = 739)**	**Quartile 3 (*n* = 716)**	**Quartile 4 (*n* = 761)**	
**Screening battery**					
Montreal Cognitive Assessment	23.6 ± 3.9	23.0 ± 4.0	22.9 ± 4.1	22.3 ± 4.2	<0.001
Digit Symbol Coding	53.1 ± 14.5	52.0 ± 15.3	50.5 ± 15.3	47.4 ± 14.7	<0.001
Logical Memory Immediate Recall	19.9 ± 4.6	19.2 ± 4.9	19.3 ± 4.9	18.7 ± 5.1	<0.001
Logical Memory Delayed Recall	8.5 ± 3.2	8.1 ± 3.4	8.2 ± 3.3	7.9 ± 3.4	0.002
**Extend battery**					
Hopkins Verbal Learning Test	28.2 ± 7.9	27.3 ± 8.1	27.1 ± 8.0	25.9 ± 8.2	<0.001
Trail Making Test A, seconds	40.6 ± 20.0	42.7 ± 25.0	45.0 ± 24.5	45.8 ± 24.2	<0.001
Trail Making Test B, seconds	115.1 ± 70.7	117.6 ± 71.3	122.8 ± 72.6	134.2 ± 75.5	<0.001
Boston Naming Test	12.0 ± 2.9	11.7 ± 3.1	11.6 ± 3.2	11.4 ± 3.4	0.01
Category Fluency—Animals	18.2 ± 5.1	18.1 ± 5.2	17.9 ± 5.1	17.0 ± 4.8	<0.001
Digit Span Total	17.2 ± 4.2	16.9 ± 4.2	16.8 ± 4.2	16.6 ± 4.1	0.018

*Values are mean ± SD.*

*Quartile 1, PP ≤ 51 mm Hg; Quartile 2, 51 mm Hg < PP ≤ 60 mm Hg; Quartile 3, 60 mm Hg < PP ≤ 70 mm Hg; Quartile 4, PP > 70 mm Hg.*

*SPRINT, Systolic Blood Pressure Intervention Trial; MIND, Memory and cognition IN Decreased hypertension; PP, pulse pressure*.

Table 3 shows the association between PP as a continuous variable and cognition domains using multiple linear regression models. PP was negatively linearly associated with the global cognition summary score in regression models adjusted for demographics [estimate (SEM): −0.03 (0.01); *P* < 0.01] and clinical characteristics [estimate (SEM): −0.05 (0.02); *P* < 0.01]. Similarly, this negative linear correlation between PP and other cognition domains including executive function, attention, memory, and language was demonstrated. In addition, we examined the association between PP and individual cognitive test (Supplementary Table 3).

**Table 3:** Association between continuous PP and summary cognition domains in SPRINT-MIND participants.

**Cognition Domains**	**PP, mm Hg (*****n*** **=3,009)**					
	**Model 1**		**Model 2**		**Model 3**	
	**Estimate (SEM)**	***P*-value**	**Estimate (SEM)**	***P*-value**	**Estimate (SEM)**	***P*-value**
Global cognition	−0.034 (0.013)	0.004	−0.054 (0.018)	0.002	−0.048 (0.018)	0.008
Executive function	−0.010 (0.005)	0.028	−0.017 (0.007)	0.014	−0.014 (0.007)	0.040
Attention	−0.008 (0.004)	0.034	−0.014 (0.006)	0.018	−0.013 (0.006)	0.035
Memory	−0.018 (0.008)	0.008	−0.024 (0.010)	0.019	−0.021 (0.011)	0.045
Language	−0.011 (0.004)	0.003	−0.021 (0.006)	<0.001	−0.020 (0.006)	0.001

*Model 1 adjusted for age, gender, race, and education.*

*Model 2 adjusted for model 1 components as well as body mass index, smoking, drinking, and cardiovascular disease.*

*Model 3 adjusted for model 2 components as well as low-density lipoprotein cholesterol, high-density lipoprotein cholesterol, fasting plasma glucose, estimated glomerular filtration rate, and medication use (statin, aspirin, and antihypertensive).*

*SPRINT, Systolic Blood Pressure Intervention Trial; MIND, Memory and cognition IN Decreased hypertension; PP, pulse pressure*.

Furthermore, we examined the association between PP and cognition involving a subset of 755 participants who had undergone brain MRI. As Supplementary Table 4 shows, there were negative linear correlations between PP and cognition domains including global cognition, executive function, memory, and language consistent in three adjustment models. No obvious linear correlation between PP and attention was observed in this study (*P* = 0.31).

### Association Between Pulse Pressure and Brain Magnetic Resonance Imaging Variables

For the MRI subgroup (*n* = 755), unadjusted performance comparisons on brain MRI variables by PP categories are shown in Table 4. PP levels and brain MRI variables including WML volume, gray matter, hippocampus, brain volume, brain lesion volume, and cerebrospinal fluid showed statistical differences. Multiple linear regression analyses indicated that PP is positively correlated with brain lesion volume [estimate (SEM): 0.03 (0.02); *P* = 0.04] and WML volume [estimate (SEM): 0.03 (0.02); *P* = 0.04]. There was no statistically significant linear association between PP and gray matter, hippocampus after adjusting for confounding factors (Table 5).

**Table 4:** Performance on brain magnetic resonance imaging (MRI) variables by PP quartile in SPRINT-MRI subgroup.

**MRI Variables**	**PP, mm Hg (n** **=** **755)**				***P*-value**
	**Quartile 1 (*n* = 234)**	**Quartile 2 (*n* = 168)**	**Quartile 3 (*n* = 179)**	**Quartile 4 (*n* = 174)**	
Intracranial volume, cm^3^	1,380.4 ± 143.5	1,393.8 ± 151.3	1,396.5 ± 146.0	1,371.9 ± 152.3	0.373
White matter volume, cm^3^	517.4 ± 55.7	521.5 ± 60.0	521.0 ± 56.0	509.6 ± 55.5	0.194
White matter lesion volume, cm^3^	2.5 ± 3.6	3.7 ± 5.9	3.5 ± 4.9	5.4 ± 7.9	<0.001
Gray matter volume, cm^3^	629.1 ± 64.7	621.3 ± 63.0	617.0 ± 63.1	590.6 ± 57.7	<0.001
Hippocampus volume, cm^3^	7.6 ± 0.8	7.6 ± 0.8	7.5 ± 0.9	7.3 ± 0.8	0.001
Cerebrospinal fluid volume, cm^3^	213.0 ± 59.6	230.2 ± 64.2	237.9 ± 61.3	251.7 ± 76.2	<0.001
Brain volume, cm^3^	1,146.5 ± 114.0	1,142.8 ± 116.9	1,138.0 ± 113.5	1,100.2 ± 107.8	<0.001
Frontal volume, cm^3^	371.5 ± 41.0	370.8 ± 42.2	367.1 ± 40.1	355.5 ± 38.6	0.001
Limbic volume, cm^3^	36.2 ± 4.3	35.9 ± 4.1	35.8 ± 4.5	34.2 ± 3.8	<0.001
Temporal lobe volume, cm^3^	214.5 ± 23.7	213.4 ± 24.2	214.5 ± 24.7	206.8 ± 22.0	0.006
Brain lesion volume, cm^3^	2.8 ± 3.6	3.9 ± 6.0	3.7 ± 4.9	5.6 ± 8.0	<0.001
Cerebral blood flow, ml/100 mg/min	33.6 ± 10.9	33.9 ± 9.5	33.4 ± 9.5	36.0 ± 11.2	0.088
Brain vascular reactivity	1.3 ± 0.5	1.2 ± 0.5	1.3 ± 0.5	1.3 ± 0.5	0.379

*Values are mean ± SD.*

*Quartile 1, PP ≤ 51 mm Hg; Quartile 2, 51 mm Hg < PP ≤ 60 mm Hg; Quartile 3, 60 mm Hg < PP ≤ 70 mm Hg; Quartile 4, PP > 70 mm Hg.*

*SPRINT, Systolic Blood Pressure Intervention Trial; MIND, Memory and cognition IN Decreased hypertension; PP, pulse pressure*.

**Table 5:** Association between continuous PP and brain MRI variables in SPRINT-MRI subgroup.

**Brian MRI**	**PP, mm Hg (*****n*** **=** **755)**					
	**Model 1**		**Model 2**		**Model 3**	
	**Estimate (SEM)**	***P*-value**	**Estimate (SEM)**	***P*-value**	**Estimate (SEM)**	***P*-value**
Brain lesion volume, cm^3^	0.033 (0.015)	0.027	0.034 (0.015)	0.025	0.031 (0.015)	0.038
White matter lesion volume, cm^3^	0.032 (0.015)	0.030	0.032 (0.015)	0.029	0.029 (0.015)	0.044
Gray matter volume, cm^3^	−0.076 (0.129)	0.559	−0.029 (0.130)	0.823	−0.067 (0.081)	0.317
Hippocampus volume, cm^3^	−0.001 (0.002)	0.555	−0.001 (0.002)	0.635	−0.001 (0.002)	0.689

*Model 1 adjusted for age, gender, race, and education.*

*Model 2 adjusted for model 1 components as well as smoking, drinking, low-density lipoprotein cholesterol, high-density lipoprotein cholesterol, fasting plasma glucose, estimated glomerular filtration rate, and medication use (statin, aspirin, and antihypertensive).*

*Model 3 adjusted for model 2 components as well as scanner type, intracranal volume, and total brain volume.*

*SPRINT, Systolic Blood Pressure Intervention Trial; MIND, Memory and cognition IN Decreased hypertension; PP, pulse pressure*.

### Association Between White Matter Lesions and Cognition

Supplementary Table 5 showed the association between WMLs and cognition. After adjusted for all covariates, WML volume was negatively correlated with cognition including global cognitive function [estimate (SEM): −0.20 (0.05); *P* < 0.01], executive function [estimate (SEM): −0.06 (0.02); *P* < 0.01], attention [estimate (SEM): −0.04 (0.02); *P* < 0.05], memory [estimate (SEM): −0.11 (0.03); *P* < 0.01], and language [estimate (SEM): −0.04 (0.02); *P* < 0.05].

### Mediation Analysis

Given the association between PP and both WML volume and cognition, mediation analysis was conducted to better understand the extent of interactions. As observed in Table 6, a fraction of cognition domain changes including global cognition, executive function, memory, and language caused by PP was partly explained by combined increases in WML volume (mediation percentage 10.8, 9.48, 10.6, and 7.2%, respectively).

**Table 6:** Mediation effect by white matter lesions (WMLs) in the association between PP and cognition in SPRINT-MRI subgroup.

	**Total effect**	**Indirect effect** **(Path A)**	**Indirect effect** **(Path B)**	**Direct effect** **(Path C)**	**Percent mediation (%)**
PP → WMLs → Global cognition	−0.095^*^ (−0.096, −0.013)	0.079^*^ (0.001, 0.058)	−0.130^***^ (−0.305, −0.091)	−0.086^*^ (−0.091, −0.008)	10.8
PP → WMLs → Executive function	−0.090^*^ (−0.036, −0.004)	0.079^*^ (0.001, 0.058)	−0.108^**^ (−0.105, −0.023)	−0.083^*^ (−0.034, −0.002)	9.48
PP → WMLs → Memory	−0.090^*^ (−0.056, −0.005)	0.079^*^ (0.001, 0.058)	−0.121^**^ (−0.175, −0.045)	−0.079^*^ (−0.052, −0.002)	10.6
PP → WMLs → Language	−0.090^*^ (−0.028, −0.002)	0.079^*^ (0.001, 0.058)	−0.082^*^ (−0.071, −0.004)	−0.085^*^ (−0.028, −0.001)	7.2

*Path A and path B together represent the indirect effect, path C represents the total and direct effects. Effects represent as β (95% confidence interval). Adjusted for age, gender, race, education, smoking, drinking, low-density lipoprotein cholesterol, high-density lipoprotein cholesterol, fasting plasma glucose, estimated glomerular filtration rate, medication use (statin, aspirin, and antihypertensive), scanner type, intracranal volume, and total brain volume.*

* *P < 0.05,*

** *P < 0.01,*

*** *P < 0.001.*

*SPRINT, Systolic Blood Pressure Intervention Trial; MIND, Memory and cognition IN Decreased hypertension; PP, pulse pressure; WMLs, White matter lesions*.

## Discussion

In a large cohort of stroke-free adults with hypertension, we confirmed that PP was negatively associated with cognition, an association mediated partly by WMLs.

There is support for the notion that patients with optimal SBP control may still have an increased risk for CVD and cognitive impairment. The impact of higher PP on target organ damage has been underestimated. In fact, higher PP as a CVD risk factor has also been shown to have a similar relationship with cognitive decline. Cognitive decline is a part of a specific hypertensive microvascular target organ damage (16). Elevated PP is a marker for increased arterial stiffness or atherosclerosis (17). Therefore, as a consequence of reduced damping of the arterial waveforms, the small vessels in the brain remodeling are exposed to high pulsating pressure. This pathological remodeling may result in impaired cerebral autoregulation accompanying endothelial dysfunction, nitric oxide synthase decrease, and oxidative stress increase, which potentially contribute to the pathogenesis and development of cerebral microvascular damage, leading to WML progression (18–22). Moreover, the Rotterdam Scan Study showed that progression of small vessel disease was paralleled with a decline in cognitive function (23). Another clinical study related arterial stiffness to cerebral WMLs (24). Furthermore, WMLs, as a marker of impaired microcirculation, increased the risk of stroke, vascular dementia, and mortality (1, 25, 26).

To demonstrate the association between PP and cognition, we summarized eight cognitive tests into five cognitive function domains, finding consistent results. Our findings extend further than most previous cross-sectional studies, relating higher PP to WMLs and lower performance on cognitive screening tests among non-stroke individuals. The negative association between PP and cognition is consistent with previous reports. In a dementia-free elderly cohort that was followed up from 0.1 to 8.3 years, higher PP was associated with an increased risk for Alzheimer disease (4). This association was confirmed in very old populations, and a study including 148 younger participants (mean age 64 years) with suboptimal BP control revealed that elevated PP during the day or night correlates with cognitive impairment (16). In addition, a U-shaped relationship between PP and cognitive decline has been observed in both healthy elderly and stroke patients (4, 8). The participants included in our study were middle-aged and older individuals aged >50 years, with an average age of 68 years, and were, therefore, broadly representative. In view of the limited sample size of participants with low PP, we did not further investigate the relationship between low PP and cognition. However, using correlation analysis, we found that participants with PP ≤ 51 mm Hg had higher cognitive test scores than those with PP > 51 mm Hg.

In previous studies, both higher SBP and DBP were strongly associated with WML severity (27–29). Kim reported for the first time that increased brachial PP is an age-independent factor associated with WMLs in asymptomatic elderly individuals^11^. This was an association that we also observed. We investigated whether brain MRI variables including brain lesion volume, WMLs, gray matter, and hippocampus are related to PP; results showed that only brain lesion volume including WMLs was positively correlated with PP, without significant correlations for the rest. Therefore, we further conducted mediation analysis to verify the hypothesis that WMLs mediate the association of PP and cognition. As a result, WMLs were found to underlie the adverse relationship between PP and multiple cognition domains, including global cognition, executive function, memory, and language.

The strengths of our study include concurrent BP measurement, brain MRI, and cognitive function tests. In addition, a large number of cognitive questionnaires were used in this study, allowing us to distinguish subtypes of cognitive deficits associated with high PP levels. Also, the study population was a large sample size of stroke-free participants with hypertension, so as to avoid the interference of stroke on study results.

## Limitations

There are several limitations in this study. First, this is a cross-sectional analysis, and the causality link between PP and WMLs cannot be inferred. Therefore, a longitudinal cohort analysis should be conducted in a further study. Second, although BP was measured using automated devices, a single BP measurement did not represent the usual BP. Thus, it is necessary to perform an ambulatory BP check to obtain the PP index. This would further extend our study results.

## Conclusions

Our study demonstrates that cognitive decline is more frequent in patients with higher PP and is related to the severity of PP. Furthermore, WMLs partially moderate the association of PP and cognition, including executive function, memory, and language. A longitudinal study should be conducted to consolidate our results and further verify the causality between PP, WMLs, and cognition.

## Data Availability Statement

The datasets presented in this study can be found in online repositories. The names of the repository/repositories and accession number(s) can be found below: https://biolincc.nhlbi.nih.gov/studies/sprint/.

## Ethics Statement

The studies involving human participants were reviewed and approved by The Ethics Review Committee of The First Affiliated Hospital of Sun Yat-sen University. The patients/participants provided their written informed consent to participate in this study.

## Author Contributions

GW and XZ provided the conception and design for the study. XZ provided the study materials or patients. JZ and JS contributed to the development of the methodology and wrote the manuscript. JL analyzed the acquired data. XZ, WW, and CY were responsible for the interpretation of statistical results. GW revised the manuscript. All authors contributed to the article and approved the final submitted version.

## Conflict of Interest

The authors declare that the research was conducted in the absence of any commercial or financial relationships that could be construed as a potential conflict of interest.
