# Supplementary material for: Pulse Pressure, Cognition, and White Matter Lesions: A Mediation Analysis
Source: Front Cardiovasc Med. 2021 May 4;8:654522. doi: 10.3389/fcvm.2021.654522 (PMC8130823; doi:10.3389/fcvm.2021.654522)
Supplement: Supplementary file 1 [file Data_Sheet_1.docx]

**Pulse Pressure, Cognition, and White Matter Lesions: a Mediation Analysis**

***Jiabin Zang^†^, Jian Shi^†^, Jianwen Liang, Xiaocong Zhang, Wenbin Wei, Chun Yao, Xiaodong Zhuang*, Guifu Wu****

**Supplementary Figure 1** 2

Flowchart of study selection

**Supplementary Table 1** 3

Tests included in the summary cognition domains in SPRINT-MIND study

**Supplementary Table 2** 4

Baseline characteristics of SPRINT-MRI subgroup participants classified by PP quartile

**Supplementary Table 3** 6

Association between continuous PP and individual tests of cognition in SPRINT-MIND participants

**Supplementary Table 4** 7

Association between continuous pulse pressure and summary cognitive function domains in SPRINT-MRI subgroup

**Supplementary Table 5** 8

Association between WMLs and summary cognitive function domains in SPRINT-MRI subgroup

**
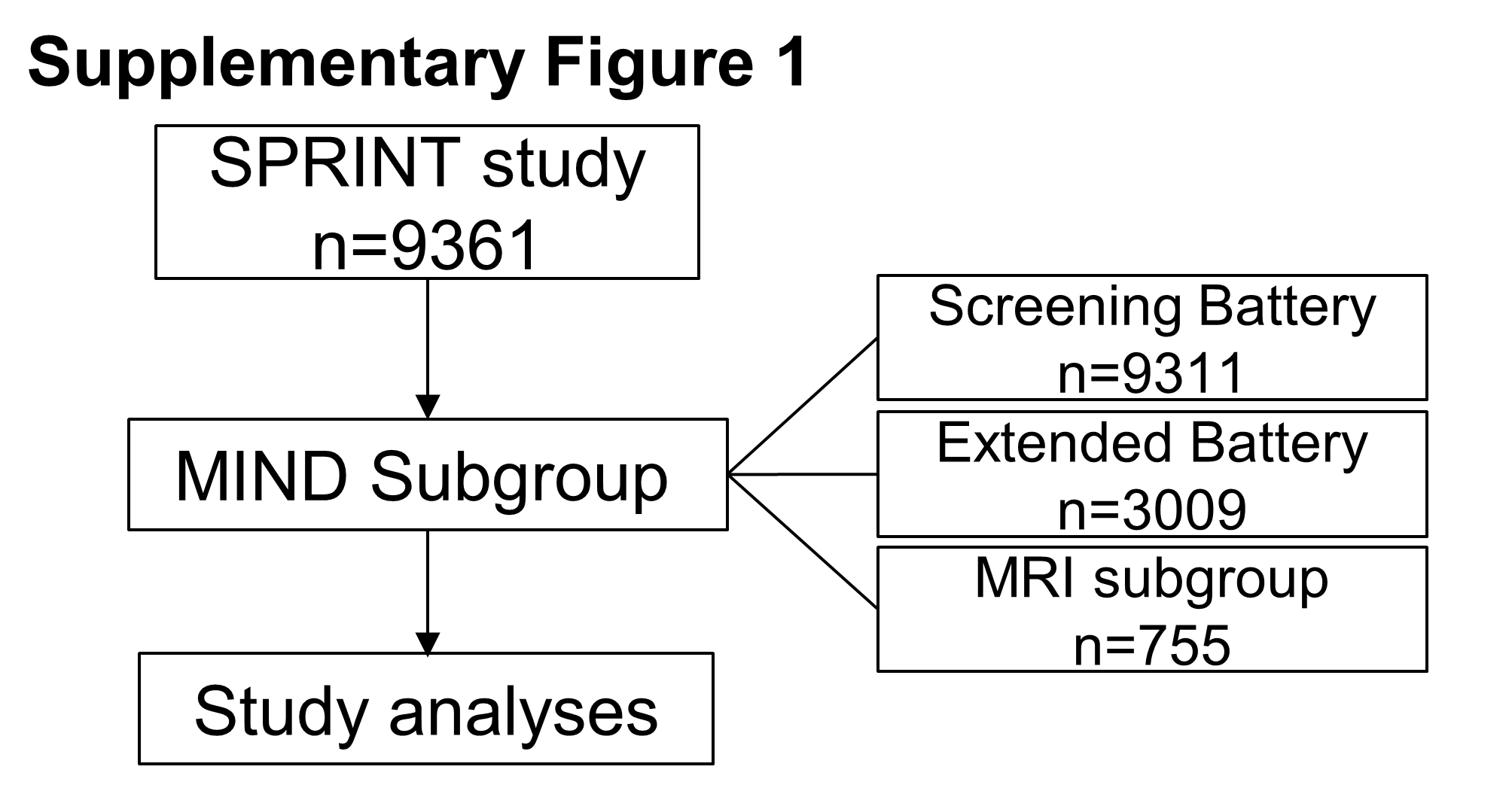
**

**Supplementary Figure 1.** Flowchart of study selection. SPRINT, Systolic Blood Pressure

Intervention Trial; MIND, Memory and cognition IN Decreased hypertension.

**Supplementary Table 1** Tests included in the summary cognition domains in SPRINT-MIND study

| Summary cognition domains | Cognitive tests |
| --- | --- |
| Global cognition | All cognitive tests in Screening^a^ and Extended^b^ battery |
| Executive function | MoCA Executive, Digit Symbol Coding, Trail Making Test B, Category Fluency Test-Animals, Digit Span Backward |
| Memory | MoCA Delayed Recall, Logical Memory Immediate and Delayed Recall, Hopkins Verbal Learning Test |
| Attention | MoCA Attention, Trail Making Test A, Digit Span Forward |
| Language | MoCA Naming and Language, Boston Naming Test |

^a^Screening battery: MoCA, Digit Symbol Coding, Logical Memory.

^b^Extended battery: Hopkins Verbal Learning Test, Trail Making Test A and B, Boston Naming Test, Category Fluency Test-Animals, Digit Span Forward and Backward.

SPRINT, Systolic Blood Pressure Intervention Trial; MIND, Memory and cognition IN Decreased hypertension; MoCA, Montreal Cognitive Assessment.

**Supplementary Table 2** Baseline characteristics of SPRINT-MRI subgroup participants classified by PP quartile

| Characteristic | Total (n=755) | PP, mm Hg | | | |  |
| --- | --- | --- | --- | --- | --- | --- |
|  |  | Quartile 1 (n=234) | Quartile 2 (n=168) | Quartile 3 (n=179) | Quartile 4 (n=174) | *P* value |
| Age,year | 68.0±8.6 | 63.2±6.8 | 67.1±7.9 | 69.2±7.9 | 73.7±8.0 | <0.001 |
| Female, n(%) | 293 (38.8) | 81 (34.6) | 59 (35.1) | 71 (39.7) | 82 (47.1) | 0.050 |
| Race, n(%) |  |  |  |  |  | <0.001 |
| White | 472 (62.5) | 133 (56.8) | 91 (54.2) | 121 (67.6) | 127 (73) |  |
| Black | 233 (30.9) | 88 (37.6) | 60 (35.7) | 46 (25.7) | 39 (22.4) |  |
| Hispanic | 36 (4.8) | 12 (5.1) | 9 (5.4) | 10 (5.6) | 5 (2.9) |  |
| Education level^a^, n (%) |  |  |  |  |  | 0.633 |
| Low | 14 (1.9) | 3 (1.3) | 3 (1.8) | 2 (1.1) | 6 (3.5) |  |
| Intermediate | 184 (24.6) | 57 (24.8) | 41 (24.4) | 48 (27.1) | 38 (22.1) |  |
| High | 549 (73.5) | 170 (73.9) | 124 (73.8) | 127 (71.8) | 128 (74.4) |  |
| Smoking status, n (%) |  |  |  |  |  | 0.020 |
| Never | 349 (46.2) | 111 (47.4) | 82 (48.8) | 69 (38.5) | 87 (50) |  |
| Former smoker | 315 (41.7) | 84 (35.9) | 68 (40.5) | 91 (50.8) | 72 (41.4) |  |
| Current smoker | 91 (12.1) | 39 (16.7) | 18 (10.7) | 19 (10.6) | 15 (8.6) |  |
| FRS, % | 19.4±10.7 | 15.1±8.1 | 17.9±8.6 | 19.7±9.8 | 26.6±12.6 | <0.001 |
| CVD history, n (%) | 107 (14.2) | 26 (11.1) | 18 (10.7) | 30 (16.8) | 33 (19.0) | 0.052 |
| CKD history, n(%) | 254 (33.6) | 63 (26.9) | 53 (31.5) | 59 (33) | 79 (45.4) | 0.001 |
| BMI, kg/m^2^ | 29.8±5.3 | 30.7±5.5 | 29.7±5.1 | 29.5±5.2 | 28.9±5.3 | 0.005 |
| SBP, mm Hg | 138.5±16.8 | 126.3±10.9 | 133.7±11.5 | 141.5±11.8 | 156.0±15.8 | <0.001 |
| DBP, mm Hg | 77.7±11.7 | 81.8±9.8 | 77.4±11.0 | 76.5±11.5 | 74.0±13.4 | <0.001 |
| LDL-c, mg/dL | 114.3±34.8 | 119.4±35.6 | 116.0±34.5 | 108.3±33.0 | 112.4±35.9 | 0.009 |
| HDL-c, mg/dL | 53.2±14.3 | 51.0±13.5 | 51.5±12.2 | 54.8±15.9 | 56.1±15.5 | 0.001 |
| Glucose, mg/dL | 98.6±13.5 | 96.9±12.6 | 99.8±12.7 | 98.5±12.1 | 99.7±15.5 | 0.090 |
| eGFR, mL/min/1.73m^2^ | 69.4±21.3 | 73.1±21.9 | 71.2±19.8 | 69.3±21.3 | 64.8±20.6 | 0.001 |
| Medication use, n (%) |  |  |  |  |  |  |
| Statin | 310 (41.6) | 85 (37.0) | 71 (42.5) | 86 (48.3) | 68 (39.8) | 0.130 |
| Aspirin | 382 (50.6) | 98 (41.9) | 81 (48.2) | 101 (56.4) | 102 (58.6) | 0.002 |
| Antihypertensive agents | 680 (90.1) | 207 (88.5) | 153 (91.1) | 161 (89.9) | 159 (91.4) | 0.753 |

Values are mean ± SD or number (%).

^a^Education level including low, below high school graduate; intermediate, high school graduate; high, beyond high school.

SPRINT, Systolic Blood Pressure Intervention Trial; MIND, Memory and cognition IN Decreased hypertension; FRS, Framingham Risk Score; CVD, cardiovascular disease; CKD, chronic kidney disease; SBP, systolic blood pressure; PP, pulse pressure; BMI, body mass index; LDL-c, low density lipoprotein cholesterol; HDL-c, high density lipoprotein cholesterol; eGFR, estimated glomerular filtration rate.

**Supplementary Table 3** Association between continuous PP and individual tests of cognition in SPRINT-MIND participants

| Cognitive tests | PP, mm Hg (n=3009) | | | | | | | |
| --- | --- | --- | --- | --- | --- | --- | --- | --- |
|  | Model 1 | |  | Model 2 | |  | Model 3 | |
|  | Estimate (SEM) | *P* value |  | Estimate (SEM) | *P* value |  | Estimate (SEM) | *P* value |
| Montreal Cognitive Assessment | -0.015 (0.005) | 0.005 |  | -0.021 (0.008) | 0.012 |  | -0.020 (0.008) | 0.018 |
| Logical Memory Immediate Recall | -0.014 (0.007) | 0.028 |  | -0.020 (0.010) | 0.042 |  | -0.019 (0.010) | 0.070 |
| Logical Memory Delayed Recall | -0.007 (0.004) | 0.116 |  | -0.009 (0.007) | 0.164 |  | -0.008 (0.007) | 0.280 |
| Digit Symbol Coding | -0.041 (0.019) | 0.035 |  | -0.015 (0.006) | 0.017 |  | -0.066 (0.030) | 0.026 |
| Hopkins Verbal Learning Test | -0.023 (0.011) | 0.030 |  | -0.028 (0.012) | 0.017 |  | -0.031 (0.017) | 0.066 |
| Trail Making Test-A Time | 0.028 (0.032) | 0.387 |  | 0.031 (0.049) | 0.521 |  | 0.020 (0.050) | 0.686 |
| Trail Making Test -B Time | 0.097 (0.097) | 0.317 |  | 0.195 (0.149) | 0.190 |  | 0.165 (0.152) | 0.277 |
| Boston Naming Test | -0.011 (0.004) | 0.006 |  | -0.028 (0.006) | <0.001 |  | -0.025 (0.006) | <0.001 |
| Category Fluency Test-Animals | -0.009 (0.007) | 0.165 |  | -0.015 (0.010) | 0.142 |  | -0.012 (0.011) | 0.251 |
| Digit Span Forward | -0.006 (0.003) | 0.071 |  | -0.010 (0.005) | 0.067 |  | -0.007 (0.005) | 0.166 |
| Digit Span Backward | -0.005 (0.003) | 0.079 |  | -0.007 (0.005) | 0.131 |  | -0.006 (0.005) | 0.189 |

Model 1 adjusted for age, gender, race and education

Model 2 adjusted for model 1 components as well as body mass index, smoking status, drinking, cardiovascular disease

Model 3 adjusted for model 2 components as well as low-density lipoprotein cholesterol, high-density lipoprotein cholesterol, fasting plasma glucose, estimated glomerular filtration rate, use of statin, aspirin and antihypertensive agents

SPRINT, Systolic Blood Pressure Intervention Trial; MIND, Memory and cognition IN Decreased hypertension; PP, pulse pressure

**Supplementary Table 4** Association between continuous pulse pressure and summary cognitive function domains in SPRINT-MRI subgroup

| Cognitive function domains | PP, mm Hg (n=755) | | | | | | | |
| --- | --- | --- | --- | --- | --- | --- | --- | --- |
|  | Model 1 | |  | Model 2 | |  | Model 3 | |
|  | Estimate (SEM) | *P* value |  | Estimate (SEM) | *P* value |  | Estimate (SEM) | *P* value |
| Global cognitive function | -0.054 (0.021) | 0.011 |  | -0.055 (0.021) | 0.010 |  | -0.054 (0.021) | 0.011 |
| Executive function | -0.020 (0.008) | 0.013 |  | -0.021 (0.008) | 0.011 |  | -0.020 (0.008) | 0.015 |
| Attention | -0.007 (0.007) | 0.297 |  | -0.007 (0.007) | 0.306 |  | -0.007 (0.007) | 0.308 |
| Memory | -0.027 (0.013) | 0.031 |  | -0.029 (0.013) | 0.026 |  | -0.030 (0.013) | 0.019 |
| Language | -0.018 (0.007) | 0.008 |  | -0.017 (0.007) | 0.012 |  | -0.015 (0.007) | 0.021 |

Model 1 adjusted for age, gender, race and education.

Model 2 adjusted for model 1 components as well as smoking, drinking, low-density lipoprotein cholesterol, high-density lipoprotein cholesterol, fasting plasma glucose, estimated glomerular filtration rate, medication use (statin, aspirin and antihypertensive).

Model 3 adjusted for model 2 components as well as scanner type, intracranal volume, total brain volume.

SPRINT, Systolic Blood Pressure Intervention Trial; MIND, Memory and cognition IN Decreased hypertension; PP, pulse pressure.

**Supplementary Table 5** Association between WMLs and summary cognitive function domains in SPRINT-MRI subgroup

| Cognitive function domains | WMLs, (n=755) | | | | | | | |
| --- | --- | --- | --- | --- | --- | --- | --- | --- |
|  | Model 1 | |  | Model 2 | |  | Model 3 | |
|  | Estimate (SEM) | *P* value |  | Estimate (SEM) | *P* value |  | Estimate (SEM) | *P* value |
| Global cognitive function | -0.164 (0.053) | 0.002 |  | -0.158 (0.054) | 0.003 |  | -0.198 (0.054) | 0.000 |
| Executive function | -0.049 (0.021) | 0.017 |  | -0.046 (0.021) | 0.029 |  | -0.064 (0.021) | 0.002 |
| Attention | -0.031 (0.017) | 0.072 |  | -0.028 (0.018) | 0.115 |  | -0.037 (0.018) | 0.043 |
| Memory | -0.088(0.032) | 0.006 |  | -0.088 (0.032) | 0.007 |  | -0.110 (0.033) | 0.001 |
| Language | -0.030 (0.017) | 0.076 |  | -0.031 (0.017) | 0.071 |  | -0.038 (0.017) | 0.028 |

Model 1 adjusted for age, gender, race and education.

Model 2 adjusted for model 1 components as well as pulse pressure, smoking, drinking, low-density lipoprotein cholesterol, high-density lipoprotein cholesterol, fasting plasma glucose, estimated glomerular filtration rate, medication use (statin, aspirin and antihypertensive).

Model 3 adjusted for model 2 components as well as scanner type, intracranal volume, brain volume.

SPRINT, Systolic Blood Pressure Intervention Trial; MIND, Memory and cognition IN Decreased hypertension, WMLs, White matter lesions.
